# A Review of Advanced Sensor Technologies for Aquatic Products Freshness Assessment in Cold Chain Logistics

**DOI:** 10.3390/bios14100468

**Published:** 2024-09-30

**Authors:** Baichuan Wang, Kang Liu, Guangfen Wei, Aixiang He, Weifu Kong, Xiaoshuan Zhang

**Affiliations:** 1 Beijing Laboratory of Food Quality and Safety, College of Engineering, China Agricultural University, Beijing 100083, China; b20193070600@cau.edu.cn (B.W.); sy20233071658@cau.edu.cn (K.L.); 2 Yantai Institute, China Agricultural University, Yantai 264670, China; 3 School of Information and Electronic Engineering, Shandong Technology and Business University, Yantai 264005, China; guangfen.wei@sdtbu.edu.cn (G.W.); or sdgshax@126.com (A.H.)

**Keywords:** aquatic products, cold chain logistics, freshness assessment, pattern recognition algorithms, freshness criteria, electronic noses, electronic tongues, biosensors, flexible sensors

## Abstract

The evaluation of the upkeep and freshness of aquatic products within the cold chain is crucial due to their perishable nature, which can significantly impact both quality and safety. Conventional methods for assessing freshness in the cold chain have inherent limitations regarding specificity and accuracy, often requiring substantial time and effort. Recently, advanced sensor technologies have been developed for freshness assessment, enabling real-time and non-invasive monitoring via the detection of volatile organic compounds, biochemical markers, and physical properties. The integration of sensor technologies into cold chain logistics enhances the ability to maintain the quality and safety of aquatic products. This review examines the advancements made in multifunctional sensor devices for the freshness assessment of aquatic products in cold chain logistics, as well as the application of pattern recognition algorithms for identification and classification. It begins by outlining the categories of freshness criteria, followed by an exploration of the development of four key sensor devices: electronic noses, electronic tongues, biosensors, and flexible sensors. Furthermore, the review discusses the implementation of advanced pattern recognition algorithms in sensor devices for freshness detection and evaluation. It highlights the current status and future potential of sensor technologies for aquatic products within the cold chain, while also addressing the significant challenges that remain to be overcome.

## 1. Introduction

Aquatic products are a critical component of the global food supply chain, providing consumers with rich nutrition and playing a key role in the global economy. However, due to their perishable nature, the freshness of aquatic products directly affects their taste and nutritional value and the health of consumers, particularly in the context of increasing foodborne diseases [1]. Therefore, maintaining freshness is crucial for ensuring product quality and safety. Over the past few decades, monitoring and protecting the freshness of aquatic products in cold chain logistics has remained a significant challenge. A stable low-temperature environment during transportation and storage is a vital factor in preventing the deterioration of aquatic products, as small fluctuations in temperature can lead to a rapid decline in freshness [2]. With advancements in science and technology, the application of advanced sensor technology is becoming increasingly important in cold chain logistics. Real-time monitoring systems based on RFID and cloud platforms not only effectively reduce the risk of quality decline but also lower logistics costs [3]. Additionally, non-destructive testing technology is critical for freshness monitoring and quality assurance in cold chain logistics [4]. Recently, advanced flexible sensor technologies have been widely adopted for freshness assessment. These flexible sensors can conform closely to the surfaces of aquatic product packaging or transportation equipment due to their lightweight and bendable characteristics. Compared to traditional sensors used for freshness assessment, the monitoring accuracy and response speed of these flexible sensors have significantly improved [5]. Notably, the development and application of advanced sensor technology have led to the increasing integration of multifunctional sensor technologies. This integration has resulted in improved efficiency in cold chain logistics and enhanced the feasibility of intelligent cold chain logistics [6]. 

The sensing technologies commonly employed for assessing the freshness of aquatic products in cold chain logistics include the electronic nose [7] and electronic tongue [8]. These sensor systems mimic the human olfactory and taste systems by integrating multiple cross-sensor arrays to detect and identify odor or liquid mixtures. The widespread application of these technologies in freshness assessment can be attributed to several key factors: (1) Rapid response capabilities [9]: They can swiftly detect volatile compounds and taste components in aquatic products, facilitating timely freshness assessments that are crucial for real-time monitoring and large-scale sample evaluation. (2) Non-destructive detection [10]: Both technologies operate without damaging the aquatic products during testing, making them suitable for continuous monitoring while preserving product integrity. As a result, the detection outcomes are reliable. (3) High sensitivity [11]: They exhibit sensitivity to low concentrations of volatile organic compounds (VOCs) and changes in the chemical composition of aquatic products. (4) Multi-parameter detection [12]: The electronic nose and electronic tongue can simultaneously detect multiple chemical components, providing comprehensive odor and taste information for a more accurate evaluation of aquatic product freshness. Despite the increasing application of non-destructive sensor technologies in freshness assessment for cold chain logistics, several limitations remain in practical applications. Firstly, when confronted with complex mixtures, these sensor technologies struggle to accurately distinguish between different source components, leading to limited recognition [13]. Additionally, the performance of electronic noses and electronic tongues may decline with prolonged use. Regular calibration and timely replacement of sensors will increase both operational costs and maintenance complexity [14]. Additionally, external environmental factors, such as fluctuations in temperature and humidity, can impact the sensor’s detection results, thereby diminishing accuracy [15]. More critically, the data analysis process is complex and challenging, necessitating the support of advanced pattern recognition algorithms [16]. 

In recent years, advancements in biological detection technologies have led to the emergence of advanced sensor devices for assessing the freshness of aquatic products, characterized by high performance and accurate sensing capabilities [17,18]. These devices integrate biological recognition elements—such as enzymes, antibodies, DNA, and cells—with physical and chemical sensors to detect specific target substances. Consequently, compared to traditional electronic noses or tongues, biosensors offer more rapid and accurate quality assessments by identifying biomarkers associated with spoilage in aquatic products, including specific metabolites, enzyme activity, and microbial content [19]. Moreover, biosensors can detect very low concentrations of biomarkers or metabolites, making them more sensitive than electronic noses and tongues in identifying incipient spoilage or minor changes [20]. Importantly, biosensors can be embedded in the packaging of aquatic products or within cold chain systems, facilitating real-time online monitoring and continuous tracking of freshness changes, which is particularly crucial during long-term transportation and storage [21]. In comparison to electronic noses and tongues, biosensors provide more comprehensive information regarding aquatic product quality [22], as they can detect multiple types of target molecules. Additionally, the data analysis process is relatively simplified, as the detection signals of biosensors often represent a direct response to specific molecules or reactions, resulting in their high specificity [23]. The emergence of flexible electronic materials and fabrication processes has facilitated the gradual development of flexible sensors characterized by their deformability and lightweight properties [24,25]. These sensors typically utilize films composed of conductive polymers, metal oxide nanomaterials, or composite materials to assess the quality of aquatic products through the detection of volatile organic compounds (VOCs) or specific gases, such as ammonia (NH_3_) and trimethylamine (TMA). In comparison to electronic noses and electronic tongues, flexible sensors offer enhanced flexibility and integrability, allowing them to be combined with various packaging materials to create smart packaging solutions that enable real-time monitoring [26]. Furthermore, the high sensitivity of flexible sensors to specific gases, such as NH_3_ and TMA, makes them particularly effective in detecting the early stages of aquatic product spoilage [27]. Additionally, flexible sensors are associated with relatively low manufacturing costs, making them easy to produce and suitable for large-scale application [28]. Notably, these sensors demonstrate considerable stability under various environmental conditions and can function effectively in harsh environments, including high humidity and elevated temperatures, thereby increasing their applicability in cold chain logistics [29,30]. 

In this review, we present the evolution and associated technologies of advanced sensor systems for assessing the freshness of aquatic products within cold chain logistics, including electronic noses and tongues, biosensors, and flexible sensors. Initially, the review outlines various freshness standards and their applications. Subsequently, we focus on the characteristics and inherent limitations of conventional freshness assessment methodologies, including electronic noses and tongues. Additionally, we introduce the principles and applications of biosensors and flexible sensors in the field of freshness assessment for aquatic products. Furthermore, we discuss four advanced sensor technologies and summarize their current advancements, highlighting their respective strengths and limitations in the freshness assessment of aquatic products within cold chain logistics. 

## 2. Traditional Indicators

**Figure 1:**
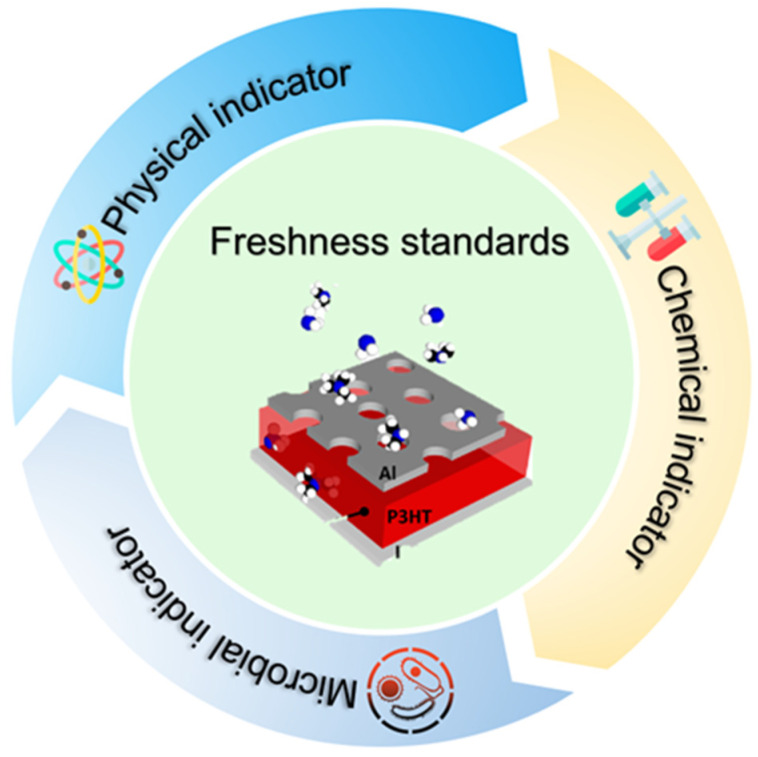
Aquatic product freshness assessment.

Due to their high-water content and rich dietary profile, aquatic products exhibit perishable traits, leading to a decline in quality and safety. Consequently, establishing a scientific framework for freshness evaluation is essential to ensure the quality of aquatic products and the safety of consumers [31,32]. Typically, as shown in Figure 1, the freshness assessment of aquatic products encompasses chemical [33], physical [34], and microbial indicators [35].

### 2.1. Chemical Indicators

Common chemical indicators include total volatile base nitrogen (TVB-N), trimethylamine nitrogen (TMA-N), and the K value [36], which reflect the extent of spoilage and quality alterations in aquatic products during storage. Among these, TVB-N is particularly notable as a primary chemical indicator for evaluating the freshness of aquatic products. It consists of nitrogen compounds such as ammonia, dimethylamine, and trimethylamine, which are generated during degradation. Goulas and Kontominas analyzed the quality changes of fish during storage and found that the TVB-N (volatile base nitrogen) content increased significantly with prolonged storage time, indicating a decline in fish meat quality [37]. Meanwhile, Oehlenschläger emphasized the importance of TVB-N as a key chemical indicator for assessing fish spoilage and freshness, particularly in cold chain logistics [38]. Additionally, TMA-N (trimethylamine nitrogen) is another widely used chemical indicator, primarily employed to detect the production of trimethylamine resulting from bacterial activity; an increase in its level is typically directly associated with the fish spoilage process [39]. In addition, Pacquit et al. achieved real-time monitoring of volatile amine compounds in the fish packaging environment by developing chromatographic sensors, including TMA and DMA, thereby providing a visual method for detecting fish freshness [40]. Dehaut et al. proposed the dimethylamine/trimethylamine ratio (DTR) as a reliable indicator of early decline in cod fillet freshness, highlighting the significance of chemical indicators in the early monitoring of fish quality [41]. 

In summary, due to their high sensitivity, chemical indicators play a crucial role in evaluating the freshness of aquatic products, particularly in cold chain logistics, where they provide detailed insights into the spoilage process. However, the detection process often necessitates complex experimental equipment and procedures, which can increase detection costs [42]. Additionally, some chemical indicators, such as total volatile base nitrogen (TVB-N), may not accurately reflect the freshness of aquatic products in the initial stages of detection, especially during the early phases of spoilage. This limitation may result in delayed responses to product quality issues and potentially compromise food safety [43]. Compared to other indicators, changes in chemical indicators are easily influenced by external environmental conditions, such as temperature and humidity, which may introduce variability in results and thus impact the reliability of detection [44]. 

### 2.2. Physical Indicators

Physical indicators are used as standards for assessing the freshness of aquatic products by measuring their physical characteristics, such as color, texture, odor, and conductivity. These indicators provide an intuitive understanding of the changes in the physical state of aquatic products throughout storage and handling. In recent years, researchers have developed various techniques to enhance the application of physical indicators in freshness evaluation. Franceschelli et al. developed non-invasive and non-destructive instrumentation techniques, such as biosensors and spectroscopic methods [45]. These methods can provide comprehensive information from a single test, enabling real-time monitoring of fish freshness during production. Özkaya and Dağbağlı investigated the use of natural pigment indicators in packaging materials, specifically for real-time monitoring of meat products [46]. These pigments, including anthocyanins and curcumin, are incorporated into smart indicator films and colorimetric sensors to assess freshness by detecting color changes associated with specific metabolites. Athauda and Karmakar introduced RFID-based sensors to monitor physical parameters such as humidity, temperature, and pH variations in smart packaging [47]. Changes in these critical physical parameters are used to evaluate the freshness of food. Ma et al. further improved the sensitivity of freshness measurement by integrating metal-organic framework hybrid matrix membranes with deep learning technology, offering a more accurate approach for real-time and integrated seafood freshness estimation [48]. Lastly, Dong et al. developed a color indicator film based on polylactic acid and lead acetate for monitoring shrimp freshness [49]. This membrane responds rapidly and precisely to hydrogen sulfide (H_2_S), with a detection limit of 0.45 ppm. The color change is linearly correlated with the freshness of the shrimp, allowing for quality assessment by the naked eye.

These physical indicators provide intuitive, rapid, and non-destructive means for assessing the freshness of aquatic products, making them well-suited for large-scale production and rapid screening. However, there are some limitations to these methods. The accuracy of these indicators is often influenced by external environmental factors such as temperature, humidity, and light, which can result in variability in evaluation outcomes. Therefore, when applying these technologies, it is crucial to consider the environmental impact on detection accuracy. Additionally, although these technologies offer real-time and comprehensive freshness measurements, integrating them into the existing food supply chain still presents challenges. Future research could explore ways to mitigate the influence of external factors and find more effective methods for integrating these technologies into practical production and supply chain processes.

### 2.3. Microbial Indicators

Microbial testing evaluates the freshness of aquatic products by measuring the total bacterial count, particularly the number of spoilage microorganisms such as Pseudomonas. Typically, an increase in the overall microbial count correlates with a decline in product quality. Moosavi-Nasab and Khoshnoudi-Nia utilized fuzzy methods to assess the shelf life of Japanese sea bream fillets [50]. Their study revealed that the shelf life of the fillets ranged from 5 to 7 days, based on microbial and chemical parameters. Wu et al. investigated the application of smart packaging technology in monitoring the freshness of fish products [19]. They incorporated indicators, sensors, and radio frequency identification (RFID) to track microbial activity within the product. Amelin et al. developed a colorimetric sensor for detecting seafood spoilage [51]. The results indicated that the sensor’s detection data aligned with measurements of the total number of microorganisms, confirming its effectiveness as a microbial indicator. Ghozzi et al. examined microbial contamination in the coastal waters of Tunisia [52]. They identified fecal indicators such as *Escherichia coli* and *Salmonella* in seawater, sediments, fish, and clams, as well as marine Vibrionaceae species, including *Vibrio alginolyticus*. Devarayan et al. created a halogen color sensor for the non-destructive measurement of spoilage in packaged fish [53]. Their experimental results demonstrated a strong correlation between the sensor readings and microbial activity, indicating its potential as a freshness indicator for aquatic products.

Compared to other indicators, microbial indicators exhibit high specificity [54] and can directly reflect the extent of spoilage processes. They also enable real-time monitoring of microbial activity, providing immediate assessments of freshness [55]. However, there are potential limitations in their practical applications. Firstly, microbial detection typically requires complex culture processes and sophisticated experimental equipment, leading to prolonged detection times, which limits their use in rapid detection scenarios [56]. Additionally, microbial growth is significantly influenced by environmental conditions such as temperature and humidity, making the detection results susceptible to external factors [57]. Although microbial indicators can provide accurate spoilage detection, they may respond slowly to early spoilage or quality degradation caused by non-microbial factors, which may not satisfy all food safety monitoring requirements [58].

In summary, while microbial indicators offer a high degree of specificity and accuracy in detecting spoilage, their practical application is hampered by the need for complex detection processes and sensitivity to environmental factors. Future research should focus on developing more rapid and reliable microbial detection methods that can be effectively integrated into food safety monitoring systems.

## 3. Development of Advanced Sensor Technologies

### 3.1. Biomimetic Sensors

#### 3.1.1. Electronic Nose (E-Nose)

In recent years, the development of advanced sensor technologies has led to the introduction of various sensor devices based on different freshness assessment standards, offering new ideas and methods for quality control in cold chain logistics. The most widely used sensor devices in practical applications are electronic noses and tongues, which mimic the human senses of smell and taste. These devices can integrate various physical and chemical detection principles to monitor the quality of aquatic products from multiple dimensions. Additionally, biosensors primarily utilize microbial detection principles to assess the freshness of aquatic products, thereby enhancing detection accuracy. The application of these technologies not only improves the accuracy and efficiency of freshness detection but also provides a scientific basis for comprehensive monitoring of cold chain logistics. Furthermore, with ongoing innovations in flexible materials and manufacturing technology, flexible sensors have emerged as a new frontier in freshness assessment applications. Importantly, the integration and application of these technologies offer robust support for quality control of aquatic products within cold chain logistics. 

**Figure 2:**
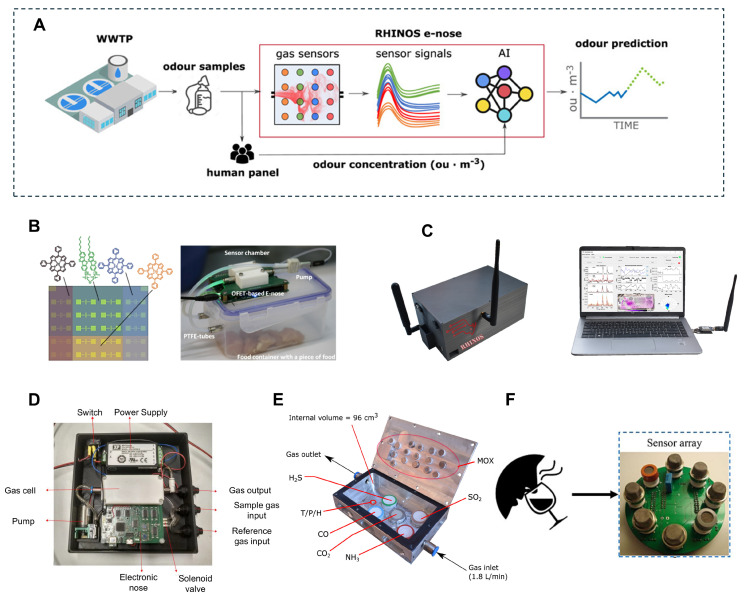
Principle and overview of electronic nose system. (**A**) Schematic representation of the RHINOS e-nose workflow for odor prediction including odor sample collection, sensor signal processing, and AI-based odor concentration prediction. Reproduced with permission [73]. Copyright 2021, Elsevier. (**B**) Schematic representation of the sensor array with 20 monolayer OFETs. Reproduced with permission [59]. Copyright 2023, American Chemical Society. (**C**) The RHINOS e-nose device and its interface with a computer for real-time data analysis. Reproduced with permission [73]. Copyright 2021, Elsevier. (**D**) The top view of a portable electronic nose based on digital and analog chemical sensors. Reproduced with permission [60]. Copyright 2022, MDPI. (**E**) RHINOS sensing chamber hosting 21 chemical sensors and a combination sensor for temperature, humidity, and pressure. Reproduced with permission [73]. Copyright 2021, Elsevier. (**F**) Schematic diagram of the electronic nose system. Reproduced with permission [74]. Copyright 2024, Elsevier.

As shown in Figure 2A, in general, the electronic nose consists of an array of gas sensors. They generate distinct electrical signals by responding to volatile organic compounds (VOC). Subsequently, the pattern recognition system utilizes techniques including artificial neural networks or principal component analysis to accurately obtain information on the freshness of the aquatic products. The working principle of an e-nose mainly involves three main steps. Firstly, the array of gas sensors composed of materials like metal oxide semiconductors, conductive polymers, or quartz crystal microbalances interacts with VOCs emitted from the aquatic products. Subsequently, each sensor in the array produces a specific response, which forms a unique “fingerprint” for each odor. Finally, the collected data are processed using pattern recognition algorithms to identify and classify the odors, including artificial neural networks, principal component analysis, and support vector machines. 

The gas sensor array is a crucial element of the electronic nose, as its performance directly influences the detection accuracy and sensitivity of the device. In order to monitor product quality and detect spoilage, as shown in Figure 2B, Daniil et al. present microbiological verification of a novel method using an electronic nose based on field-effect transistors [59]. In addition, the portable real-time and high-speed electronic nose for odorous compounds was designed (Figure 2C). Melendez et al. introduced a novel prototype of an electronic nose using an array of digital and analog metal-oxide sensors with 31 signals, and the effectiveness of responding to low concentration in laboratory conditions was verified (Figure 2D) [60]. Radi et al. developed a system based on the electronic nose and measured the freshness of tilapia using the normalized absolute data method, achieving an accuracy of 93.88% [61]. As technology continues to advance, the classification capabilities and prediction accuracy of electronic noses have also been improved. Sanchez et al. developed a prediction model through discriminant neural network analysis and the partial least squares method to classify the freshness of fish and predict microbial parameters, with a success rate of 85% [62]. In addition, Grassi et al. developed a portable simplified electronic nose system called Mastersense for assessing the freshness of meat and fish and achieved high-precision classification results [63]. In order to meet the needs of modern food preservation, electronic nose technology is gradually integrated with Internet of Things technology. For example, Wang et al. developed a mini electronic nose system based on the Internet of Things platform for real-time assessment of the freshness of food in the refrigerator, showing its high efficiency in identifying fresh, semi-fresh, and spoiled aquatic products [64]. At the same time, Wijaya et al. used an electronic nose combined with machine learning algorithms to propose a fast, low-cost, and accurate seafood quality detection method and showed extremely high accuracy in classification tasks [10]. In recent studies, electronic nose technology has been used to assess changes in volatile compounds in processed foods. For example, Alloyarova studied the application of the electronic nose in the assessment of volatile compounds in smoked semi-finished capelin, revealing the impact of different processing methods on the composition of compounds [65]. In addition, Madhubhashini et al. developed a portable electronic nose system for assessing tuna freshness and achieved high-precision storage days classification through odor sampling and volatile base nitrogen analysis [66].

The pattern recognition system is also a crucial component of electronic nose technology, primarily tasked with extracting features from the complex signals acquired by the sensor array to classify or predict the freshness of aquatic products. Recent research has been immerged in artificial intelligence and machine learning and has led to significant progress in the processing of complex odor data by pattern recognition systems for electronic noses. Gholamhosseini et al. used electronic nose technology and an intelligent information processing system to successfully identify the number of days fish have been kept after being caught, with an accuracy of 91% [67]. Tian et al. used an electronic nose system based on principal component analysis (PCA) to quickly classify the freshness of fish and pork. The results showed consistency with standard freshness indicators such as TVBN and aerobic bacterial count [68]. With the development of computing technology, more advanced pattern recognition algorithms are applied to electronic nose systems. For example, Sun and Zhang combined a convolutional neural network (CNN) with a gas sensor array system to successfully predict the freshness of refrigerated tilapia, with a prediction accuracy of 92.31% [69]. In addition, Zhu et al. used an electronic nose system based on the Laplacian eigen mapping (LE) method to accurately classify three quality levels of Chinese mitten crab (fresh, semi-fresh, and spoiled), showing the system’s potential in aquatic product quality assessment in cold chain logistics [70]. Other studies have also shown that integrating multiple sensing technologies can significantly improve the performance of electronic nose systems. In addition, Weng et al. integrated the electronic nose with computer vision and artificial tactile sensing technology to greatly improve the effect of meat freshness detection [71]. Furthermore, Mohareb et al. demonstrated that combining an electronic nose with an ensemble support vector machine classifier (SVM) can significantly improve the prediction accuracy of beef quality assessment [72]. 

As shown in Table 1, the convolutional neural network has the most outstanding performance, with an accuracy of 92.31% and a sensitivity of 91.5% due to its strong ability to process complex and non-linear data, which makes it more effective in freshness prediction of aquatic products. However, its high computational complexity increases model training time. In contrast, although principal component analysis, as a traditional statistical method, has an accuracy of 91% and a sensitivity of 90.9%, it easily processes data and can quickly respond. Thus, the PCA method is particularly suitable for processing data of multiple types of aquatic products. However, it is sensitive to noise and may not perform well when processing small sample data. As a nonlinear dimensionality reduction method, when faced with complex data, the accuracy of the LE model is 85%, and the sensitivity is 84.1%, which is slightly lower than other methods. In addition, due to the high complexity of the algorithm, LE also requires large computing resources, which may affect its widespread promotion in practical applications. Compared with other models, integrated SVM enhances the robustness of decision-making by combining multiple SVM classifiers, with an accuracy and sensitivity of 84.1% and 72.7%, respectively. Although its accuracy is slightly lower than PCA and CNN, SVM has significant advantages in handling high-dimensional data, especially when dealing with data sets with high diversity. However, the SVM method is sensitive to parameter selection and has large computational cost. The UFGC method performed well in the freshness assessment of frozen and refrigerated foods, with an accuracy of 89% and a sensitivity of 88%. The main advantages of UFGC are its fast detection speed and high resolution, making it very suitable for rapid detection of meat and aquatic products. However, the potential limitation of this method is that the equipment is expensive, and the operation is complex. Therefore, when requiring high accuracy and sensitivity, convolutional neural networks are the better choice. When requiring fast processing and analysis, PCA and UFGC methods can show better performance. Furthermore, LE and integrated SVM methods are suitable for specific complex data analysis tasks.

The advancements in electronic nose technology have greatly enhanced its capability to accurately detect spoilage and assess the freshness of various food products. With the integration of IoT and machine learning algorithms, these systems have become more efficient and versatile, enabling real-time monitoring and precise classification. However, while electronic noses have shown promising results, there are some challenges to consider. First, the reliance on gas sensor arrays means that the device’s accuracy can be affected by environmental factors such as temperature, humidity, and interference from other volatile compounds. Calibration and standardization of these sensors are necessary to ensure consistent performance. Furthermore, while integrating electronic noses with IoT platforms offers improved monitoring capabilities, it also requires a robust data management system to handle and analyze the large amounts of data generated.

Future research should focus on refining the technology to minimize environmental influences, enhancing the algorithms used for classification and prediction, and developing more user-friendly interfaces for broader application in the food industry. Additionally, exploring the use of electronic noses in combination with other freshness assessment methods could provide a more comprehensive approach to food quality monitoring.

#### 3.1.2. Electronic Tongue (E-Tongue)

**Figure 3:**
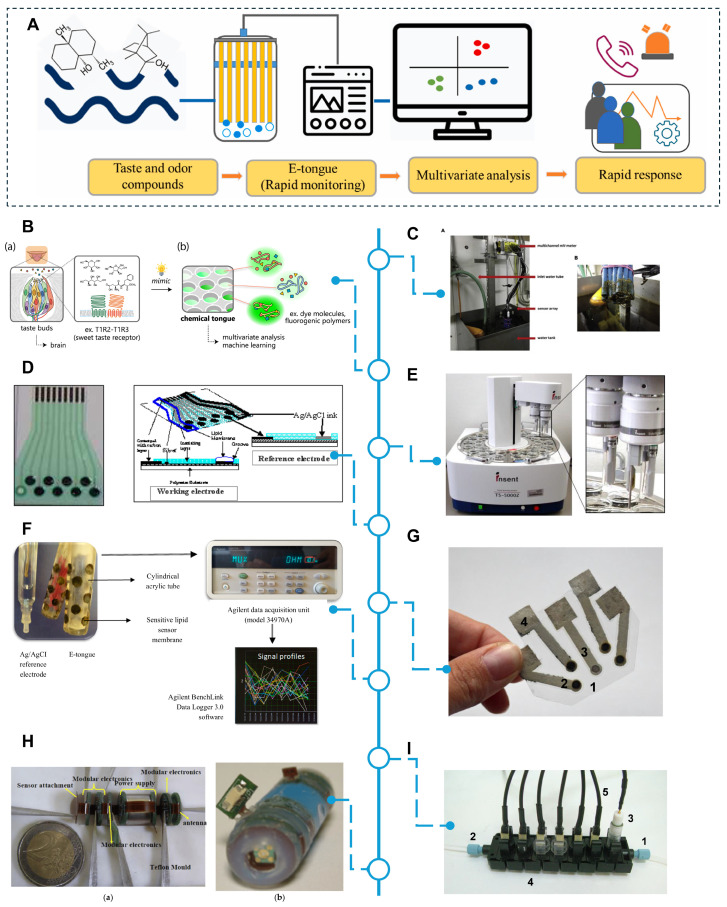
Principle and overview of electronic tongue system. (**A**) Schematic representation of the E-tongue workflow for odor and taste determination. Reproduced with permission [87]. Copyright 2023, Elsevier. (**B**) Schematic representation of the chemical tongue with a representative element (fluorogenic polymer). Reproduced with permission [88]. Copyright 2022, Springer Nature. (**C**) Photo of the potentiometric E-tongue used for the on-line measurement at an aeration plant and detail of the sensors. Reproduced with permission [89]. Copyright 2022, Elsevier. (**D**) The front view and cross-sectional view of electronic tongue sensor strip. Reproduced with permission [90]. Copyright 2008, MDPI. (**E**) TS-5000Z taste sensing system, Intelligent Sensor Technology Inc., Kanagawa, Japan. Reproduced with permission [91]. Copyright 2018, MDPI. (**F**) Lab-made E-tongue device: sensor arrays, data logger, and control software installed on a PC [92]. Copyright 2021, MDPI. (**G**) Paper-based electronic tongue system applied for discrimination. Reproduced with permission [91]. Copyright 2018, MDPI. (**H**) Sensor matrix in the form of a swallowable capsule. Reproduced with permission [91]. Copyright 2018, MDPI. (**I**) Example of an electronic tongue system with individual sensing modules applied for the analysis of fermentation: 1. Inlet, 2. Outler, 3. Reference electrode, 4. Single modules with individual sensors, 5. Electronic connections. Reproduced with permission [91]. Copyright 2018, MDPI.

Electronic tongue [74,75,76,77,78] technology utilizes chemical sensor arrays to simulate the human taste system (Figure 3A), enabling the evaluation of aquatic product freshness through the detection and analysis of chemical components in liquids. Kaya et al. emphasized that electronic tongues are widely used in the food industry to evaluate the freshness and maturity of fruits, vegetables, meats, beverages, and dairy products, and their objectivity is better than traditional subjective taste analysis [79]. The study by Munekata et al. summarized the application of electronic tongues in the quality, safety, and shelf-life monitoring of meat and aquatic products, pointing out that electronic tongues perform well in rapid sensory profile assessment and quality inspection in these areas [11]. Cho et al. further pointed out that the significant correlation between the electronic tongue and human sensory data makes it a powerful tool for sensory evaluation and quality inspection [80]. The study by Li et al. demonstrated the application of the electronic tongue in detecting counterfeit green tea, using sensory evaluation and pattern recognition methods, such as principal component analysis (PCA) and hierarchical cluster analysis (HCA), and achieved satisfactory results [81]. The electronic tongue developed by Piccinin et al. can detect lactose content in commercial foods, demonstrating the potential of this technology in food quality assessment [82]. In the field of aquatic products, Duan et al.’s study pointed out that the application of engineered nanomaterials (ENMs) in assessing the freshness of meat and aquatic products has demonstrated its effectiveness as a freshness assessment tool, but at the same time, the use of nanomaterials has risks in the food industry [83]. The study by Nowshad et al. reviewed the application of electronic tongues in food safety and quality assessment, emphasizing its importance in the freshness assessment of aquatic products [84]. The wireless pH sensor technology developed by Waimin et al. provides a low-cost solution for the assessment of microbial spoilage risks of aquatic products, which has great application potential throughout the supply chain [85]. The research by Cong et al. demonstrated an on-site freshness assessment method based on MnO2 nanosheets, which was combined with the WeChat applet to achieve highly sensitive on-site assessment [86].

Compared with the conventional electronic nose, although both simulate the human sensory system to detect and assess the freshness of aquatic products, there are differences in detection mechanisms. The electronic tongue mainly simulates the human taste system and focuses on the taste components in liquid samples, while the electronic nose simulates the human olfactory system and detects volatile organic compounds in gas samples. In addition, electronic tongues and electronic noses can generate multi-dimensional data. Complex aquatic products can be classified and predicted by pattern recognition and statistical analysis. However, electronic tongue devices are usually more complex due to more sensor data and processing steps, which will increase the application costs. Furthermore, electronic tongue devices have relatively slow response speeds due to the involvement of liquid handling and multi-step detection, which increases the detection time.

### 3.2. Biosensors

Currently, the freshness assessment of aquatic products using biosensors devices have rapidly developed. Compared with the electronic nose and tongue, biosensors [93,94,95,96,97,98] detect chemical or biological markers in aquatic products through specific biological components (such as enzymes, antibodies, DNA, etc.) recognition to provide more direct and specific detection results. Thus, it has high sensitivity and specificity. For example, Chen et al. developed a fluorescent biosensor based on platinum nanoparticles for the detection of hypoxanthine in aquatic products. Its sensitivity and selectivity are superior to traditional electronic nose and electronic tongue methods [99]. In addition, biosensors also can quickly detect specific biomarkers in aquatic products, such as histamine or hypoxanthine, which are important indicators of the freshness of aquatic products [100]. 

Differing from traditional electronic noses and tongues, biosensors typically consist of three main components: biorecognition elements, converters, and signal processing systems. Firstly, the biorecognition elements, including enzymes, antibodies, nucleic acids, or cells, interact specifically with the target analyte. Then, the converter then transforms this biological reaction into a measurable physical signal, such as an electrical, optical, or thermal signal. Finally, the signal processing system amplifies, processes, and displays these signals as readable output. 

**Figure 4:**
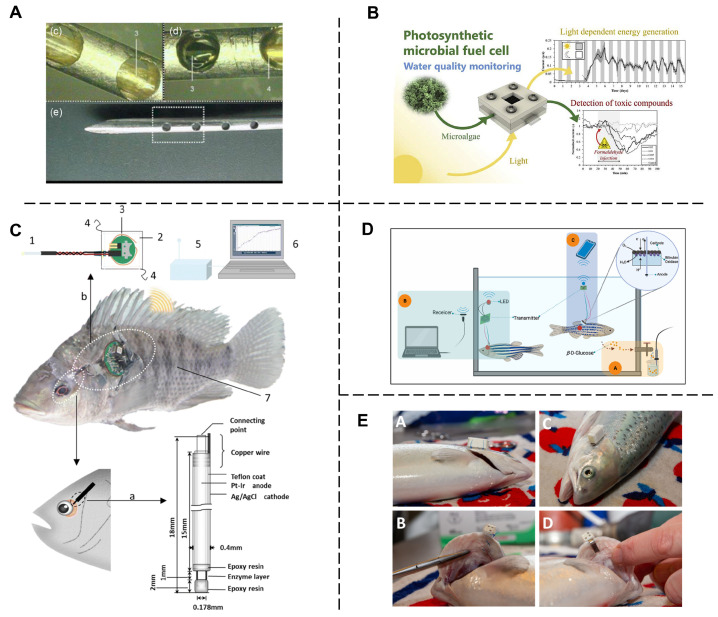
Overview of biosensors application. (**A**) Schematic diagram of the needle-type enzyme sensor system and images of the detector region. Reproduced with permission [111]. Copyright 2019, Springer Nature. (**B**) Schematic representation of the operation of light-dependent bio-electrochemical systems [112]. Copyright 2019, Elsevier. (**C**) Schematic diagram of the wireless biosensor system for fish. 1 Needle-type enzyme sensor, 2 waterproof sheet, 3 wireless potentiated, 4 nylon threads, 5 receiver, 6 personal computer, 7 test fish (Nile tilapia *Oreochromis niloticus*) [111]. Copyright 2019, Springer Nature. (**D**) Schematic diagram of biosensor for detecting the blood glucose of live fish [113]. Copyright 2022, Elsevier. (**E**) AEFishBIT biosensor attached to Atlantic salmon (*Salmo salar*) operculum using surgical thread and self-piercing fish tag [114]. Copyright 2021, MDPI.

In recent years, research on the application of biosensors for assessing the freshness of aquatic products has made significant progress. An electrochemical biosensor based on a copper-based metal-organic framework nanofiber membrane was developed by Wang et al., which detects hypoxanthine and xanthine in refrigerated seafood by immobilizing xanthine oxidase [101]. Experimental results show that the sensor exhibits high sensitivity and good recovery rate in detecting the freshness of seafood, showing its great potential in practical applications. Liu et al. proposed an irreversible sensor for the deterioration of protein foods (including seafood and meat) to cope with the need for detection of food degradation during storage [102]. The application of this sensor can help prevent food processing companies from committing fraud related to product quality, thereby improving food safety. Luo et al. developed a chromatographic indicator array for the detection of volatile amines during seafood spoilage [103]. This dye-based indicator can determine the freshness of food by detecting the presence of volatile amines during the spoilage process of seafood and has application prospects. Research conducted by Franceschelli et al. shows that the application of biosensors for fish freshness detection overcomes the limitations of traditional methods and provides non-invasive and non-destructive online process control options without the need for complex sample preparation [45]. Milintha Mary et al. developed a dual sensor based on a pH indicator to detect the freshness of packaged seafood by monitoring color changes, which is an effective tool for evaluating the quality of seafood [104]. González-Martín et al. developed an array-based sensor for the assessment of seafood freshness [105]. The sensor uses conductive polymer elements and custom electronics to detect small signal changes, enabling assessment without microbial counting technology. Electrochemical biosensors [106] have shown significant advantages in the quantitative detection and screening of food contaminants, especially in ensuring the freshness of seafood. In addition, colorimetric indicators [107] stimulated by volatile alkaline nitrogen have become an effective tool for monitoring the freshness of seafood due to their obvious color changes, promoting real-time detection and consumer understanding. Wijaya et al. uses electronic nose and machine learning algorithms to evaluate the freshness quality of marine fishery products (seafood) through classification and regression tasks, achieving high-precision detection and being able to identify microbiota [10]. A ciELISA was developed by Sheng et al. for rapid, specific, and accurate detection of tyramine in meat and seafood samples as a useful tool for assessing the freshness of protein foods [108]. Chen et al. developed a halochromic label based on a cationic guar gum and kappa carrageenan hybrid packaging label, capable of dynamically monitoring the freshness of shrimp through three identifiable hue stages (fresh, spoilage, and spoilage) [109]. Mary et al. developed a smart film based on butterfly pea anthocyanins and TiO_2_, which can be used as a freshness sensor for shrimp during storage, changing color over time to indicate spoilage [110]. In addition, as shown in Figure 4A, for rapid and simple determination of glucose concentrations in fish blood, a biosensor with a hollow needle-type container and optical oxygen fiber probe with a ruthenium complex was developed [111]. Chouler et al. used microalgae in microbial fuel cells (MFCs) as a means to generate a sensitive, portable, and cost-effective bio electrochemical sensor for onsite monitoring of pollutants in water (Figure 4B) [112]. Hideaki et al. developed a novel wireless biosensor system to monitor the concentration of fish and can obtained real time measurements of blood glucose concentrations even while test fish swim freely (Figure 4C). As shown in Figure 4D, a current-based biosensor with the glucose oxidase enzyme (GOx) immobilized on a semi-permeable polyester membrane was designed to get low concentrations of *β*-D-glucose of farmed fish. A novel miniaturized biosensor was present to monitor Atlantic salmon swimming activity and respiratory frequency. 

The main reasons why biosensors are widely used in freshness assessment of aquatic products are as follows: (1) biosensors show high sensitivity and high accuracy due to their specificity. (2) Biosensors have significant advantages in detection speed and portability because they are usually designed as portable devices suitable for real-time detection in the field. In addition, their quick response can prevent product quality by detecting the freshness of aquatic products in time. (3) Biosensors are more straightforward in terms of data analysis and result interpretation. Because it detects specific target molecules, the interpretation of the results is relatively simple and clear, and there is no need to rely on complex pattern recognition algorithms to analyze multi-dimensional data like electronic noses and electronic tongues, which makes the biosensor more intuitive in operation and result analysis. 

### 3.3. Flexible Sensors

Flexible sensors that can maintain functionality under various deformations and stresses are widely used in fields such as flexible electronic devices, biomedical engineering, and food quality monitoring. In recent years, with the increasing importance of cold chain logistics in the aquatic product supply chain, the application of flexible sensors in aquatic product freshness assessment has received significant attention and research due to their high sensitivity, attachability, and portability [115,116,117,118,119,120]. 

**Figure 5:**
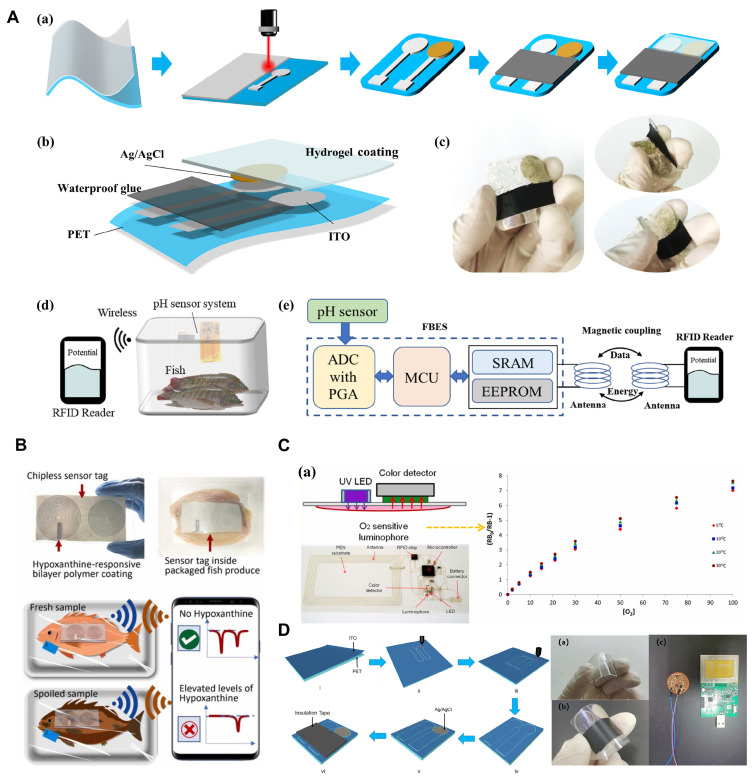
Overview of biosensors application. (**A**) Diagram of design layout and schematic of the hydrogel coating flexible pH sensor system for TVB-N monitoring. Reproduced with permission [132]. Copyright 2022, Elsevier. (**B**) Schematic representation of the electronic-free and low-cost wireless sensor tag for monitoring fish freshness [133]. Copyright 2023, Elsevier. (**C**) Schematic diagram of A screen printed flexible RFID tag for O_2_ monitoring [134]. Copyright 2023, Elsevier. (**D**) Schematic diagram of flexible wireless pH sensor system based on ITO coated flexible PET substrate for fish quality detection [133]. Copyright 2023, Elsevier.

Flexible sensors are usually composed of flexible base materials, conductive materials, and sensitive layers. Base materials such as polyimide (PI), polydimethylsiloxane (PDMS), etc., give the sensor high flexibility and durability. In addition, conductive materials such as silver nanowires, carbon nanotubes, etc., ensure the electrical signal of the sensor. The sensitive layer is used to determine the detection performance of the sensor, such as improving the response to specific gases or chemical substances through functionalization. Currently, some flexible sensors have been reported. González-Martín et al. developed an array-based sensor for evaluating the freshness of aquatic products, which combines a chemical sensor array and custom electronics to allow the freshness of seafood to be assessed without the need for microbial counting technology [105]. Pacquit et al. developed a colorimetric sensor to monitor amine compounds produced by fish spoilage in packaging and achieved real-time monitoring of the freshness of aquatic products through color changes [40]. Andre et al. studied a tag system that uses flexible sensors combined with wireless radio frequency communication technology to achieve real-time monitoring of food quality by detecting volatile amine compounds in aquatic product samples [30]. Waimin et al. developed a low-cost, non-reversible wireless pH sensor that can detect spoilage in packaged meat products, demonstrating its potential application in freshness detection of aquatic products [64]. Mary et al. designed a starch smart film based on butterfly pea anthocyanins and TiO_2_, which can indicate the spoilage of shrimp during storage through color changes [111]. Mahajan et al. developed a small flexible sensor respirometer that can measure the respiration rate, respiratory quotient, and hypoxic limit of fresh agricultural products in real time, showing its application potential in aquatic product freshness detection [121]. Yilmazoğlu developed a digital image colorimetric biosensor that demonstrated extremely high selectivity and sensitivity by utilizing TLE/PEG/Ag/AgO nanoparticles for real-time quantitative detection of hydrogen peroxide [122]. Mestry et al. studied a pH sensor developed using imine-azo dyes derived from vanillin and salicylaldehyde. Although the research focus was not on freshness detection of aquatic products, it demonstrated the potential of smart packaging [123]. Dudnyk et al. developed an edible sensor based on pectin and red cabbage as a colorimetric indicator of the freshness of aquatic products. It showed high sensitivity to amine gases and performed well in actual food samples [124]. Wu et al. designed a seafood freshness detector based on the TGS2603 gas sensor module and STM32 microcontroller system, which helps determine the freshness of aquatic products by quickly measuring the trimethylamine gas released from food [125]. Konoplev et al. developed a new chromatographic optical sensor to rapidly assess the freshness of aquatic products and poultry based on ATP metabolite content through deep ultraviolet LED photometric detection, providing a more cost-effective and efficient alternative to traditional methods [126]. The flexible sensor studied by Huang et al. combined with a machine learning algorithm can instantly detect the freshness of mutton, with an average prediction accuracy of 91.63%, which is better than the traditional Arrhenius equation method [115]. Li et al. developed a flexible ammonia sensor based on PEDOT/silver nanowire composite film to monitor the freshness of meat, demonstrating its feasibility in early meat quality detection [127]. Jia et al. studied cellulose-based radiometric fluorescent materials, which can be used to detect the freshness of shrimps and crabs in real time and visually [28]. Zhao et al. designed a sensor based on gold/WO_3_ nanosheets that can efficiently and selectively assess the freshness of aquatic products by detecting trimethylamine gas [20]. Zhang et al. developed a highly sensitive trimethylamine QCM sensor based on porous functionalized tungsten disulfide/polyacrylic acid composite for freshness detection of aquatic products, showing better selectivity compared with single-material sensors. and response time [128]. Zeng et al. studied a hydrogel based on polyvinyl alcohol/chitosan to achieve naked-eye real-time monitoring of the freshness of aquatic products through color changes related to volatile basic nitrogen (TVB-N) and pH [129]. Choi et al. developed an NFC-enabled dual-channel flexible sensor tag that combines resistive and capacitive readout channels, which can be used to monitor the freshness of seafood products by tracking temperature and ethylene concentration in the storage environment [130]. The study by Zhu et al. demonstrated the application of flexible sensors in detecting shrimp freshness and further verified its effectiveness in evaluating the freshness of aquatic products [24]. Kim et al. developed a dye-functionalized sol-gel matrix combined with carbon nanotubes to create a refreshable and flexible gas sensor to achieve selective detection of harmful gases such as NH_3_, Cl_2_, and SO_2_ [131]. As shown in Figure 5A, a hydrogel coating flexible pH sensor system was described for real-time wireless monitoring of fish spoilage by Mu et al. Sarath et al. present an electronic-free wireless sensor tag that can selectively monitor hypoxanthine (HX) as a specific biomarker to indicate the spoilage of fish products (Figure 5B). With the development of flexible material and fabrication, flexible gas sensors have been designed with a screen-printed flexible RFID tag for O_2_ monitoring in food packaging and quality actual monitoring application, which is deposited on the inner surface of flexible PEN substrate (Figure 5C). In addition, Mu et al. proposed a flexible wireless pH sensor system based on indium tin oxide (ITO) coated flexible PET substrate for achieving fish quality detection (Figure 5D).

The main advantages of flexible sensors are their high sensitivity, customizability, and mechanical flexibility. Because they can be directly attached to the surface of aquatic products or packaging materials, flexible sensors can provide more accurate and real-time monitoring data than traditional rigid sensors. In addition, the bendable and stretchable properties of flexible sensors enable wider adaptability in complex packaging environments. Although flexible sensors have shown great potential in aquatic product freshness assessment, their application still faces some challenges. First, the stability and durability of flexible sensors are limited by material properties, especially in aquatic product cold chain logistics. Frequent changes in temperature and humidity may accelerate material aging. Secondly, the manufacturing cost of flexible sensors is high, especially in large-scale production and application, which may limit their application in a wide range of markets. In addition, since flexible sensors usually need to be integrated with other sensing technologies, such as wireless transmission modules and data processing systems, the complexity and power consumption of their systems are also technical difficulties that need to be overcome.

## 4. Comparison and Application of Sensor Technologies

As shown in Table 2, the reason why electronic nose technology is widely applied in aquatic quality and freshness detection is its short response time and ability to perform fast monitoring with minimal preprocessing. Furthermore, it has a wide evaluation range and offers good repeatability, making it suitable for rapid odor detection in food products. However, the electronic nose is highly susceptible to environmental interference, such as changes in temperature and humidity, which can affect its accuracy. Additionally, the high equipment cost is a significant limitation, restricting its widespread adoption. In addition, the electronic tongue can detect low concentrations of substances generated in the early stages of food spoilage or contamination. This makes it valuable for early taste detection, ensuring that food quality can be monitored promptly. Despite this advantage, it shares similar disadvantages with the electronic nose, including susceptibility to environmental interference and high equipment costs, which can hinder its routine use in various settings. For biosensors technology, it is suitable for precise biochemical analysis in controlled conditions due to high sensitivity and specificity. They can detect target molecules at very low concentrations, which is crucial for applications requiring high precision. However, biosensors suffer from poor stability, particularly when used in environments that vary in conditions. This lack of stability limits their applicability in continuous and long-term freshness monitoring. This emerging flexible sensor technology offers flexibility and multi-functional integration, which are key for real-time and continuous monitoring of food freshness, particularly in aquatic products. Flexible sensors can conform to various shapes and surfaces, allowing for more comprehensive and adaptable monitoring. Despite these promising features, flexible sensors currently face challenges regarding stability and long-term use in complex environments. Ongoing advancements in material science and manufacturing are expected to improve their performance and reliability.

In summary, although each sensor technology has its distinct advantages and disadvantages, their specific application areas highlight their unique contributions to food quality assessment. Electronic noses and tongues are best suited for rapid detection scenarios, biosensors for precise biochemical analysis, and flexible sensors for real-time, adaptable monitoring. Understanding these differences is crucial for selecting the appropriate sensor technology for specific food monitoring needs.

## 5. Discussion and Future Prospects

More recently, with the advancement of science and technology, electronic nose and electronic tongue technologies have made significant developments in the field of food freshness testing. In particular, the integration of Internet of Things (IoT) technology and portable electronic noses based on organic field effect transistors (OFETs) greatly improves the accuracy and efficiency of fish quality monitoring. Anisimov et al. presents a portable electronic nose based on organic field-effect transistors [59]. These results show that it not only provides fast and accurate detection but also has a low production cost, suitable for large-scale application, especially in different aspects of cold chain logistics. Furthermore, these devices are typically small and lightweight, making them suitable for mobile inspection during transport and storage, providing flexible monitoring and control. In addition, IoT technology has been widely used in the expansion of devices including electronic noses and tongues due to the ability for real-time monitoring and feedback. Damdam et al. developed a versatile IoT-enabled electronic nose system to monitor food quality by evaluating the concentrations of volatile organic compounds [135]. IoT systems are capable of automatically recording and analyzing sensor data, as well as analyzing and predicting it through intelligent algorithms. This significantly reduces the need for manual testing and improves monitoring efficiency. For biosensors, a number of new technologies have been integrated in them. With the development of microelectronic components, electronic nose and tongue systems are expected to be integrated into household appliances or smart packaging, or even become standard equipment for daily food testing. Compared with conventional sensor technologies, nanotechnology sensors [136] perform better in terms of detection selectivity, sensitivity, and detection limits, enabling more accurate monitoring of freshness, in particular, suitable for real-time inspection in the food supply chain. In Vu et al., an immunosensor-based real-time monitoring technique was developed for continuous monitoring of small molecules in food processing [137]. The sensor is capable of detecting toxic alkaloids in potato juice within 5 min and can operate continuously for 20 h with a high degree of sensitivity and rapid response. In addition, smart biosensor packaging technology makes it easier and more efficient to check food freshness [138]. By using smart packaging, consumers and all parties in the supply chain can access information about the freshness of food directly through sensors, reducing waste and improving food safety. A fiber optic-based biosensor technology was designed for rapid detection of pathogenic bacteria in foods [139]. With high sensitivity and real-time detection capability, this technology is suitable for pathogenic bacteria detection in food and agricultural products and can significantly improve the efficiency of food safety detection. Therefore, fiber optic biosensors provide a new pathogenic bacteria detection method for the food industry, which has a wide range of application prospects, especially suitable for on-site detection in food production. The future of biosensors will focus on multifunctionality and miniaturization, which are combined with nanotechnology and multi-sensor integration to allow the development of systems that detect multiple volatile compounds simultaneously. In addition, these sensors will be increasingly used in all parts of the food supply chain, from production to final consumption, to improve the ability to monitor food freshness in real time and reduce food waste and improve food safety. With ongoing advancements in flexible sensor technology, including improvements in material properties and micro-nano fabrication techniques, these sensors are anticipated to become more stable and reliable. Their application in real-time quality assessment of aquatic products could revolutionize the industry, providing more accurate and continuous data on product freshness. Li et al. summarized how flexible organic/polymer gas sensors are used in smart packaging and strategies for integrating gas sensors with packaging systems [140], which provide a new means of monitoring smart packaging, reducing food waste and improving food safety. Zhu et al. developed a flexible ammonia sensor using multi-walled carbon nanotubes and PDMS films to test its application in breath analysis and food safety, which shows high sensitivity to NH3 at room temperature and also excels in detecting shrimp freshness [24]. Importantly, it is particularly suitable for inspection in high humidity environments. Besides, Xia et al. present a flexible temperature/impedance sensing patch based on graphene material for temperature monitoring and freshness classification during fish storage [141]. The patch is interference-free in high humidity environments and features high-precision freshness classification with 93.07% accuracy. Moreover, this low-cost, customized flexible sensor provides a practical solution for fast, non-destructive detection of food quality. In the future, flexible sensors will have great potential for customizable, integrated smart packaging and mobile device applications. The developments will focus on reducing manufacturing costs and improving the sensitivity, durability, and reproducibility of sensors. At the same time, the deep integration of flexible sensors with the Internet of Things (IoT) is also expected to make them a tool for daily food monitoring for consumers, driving personalized food freshness monitoring. In conclusion, the Internet of Things and artificial intelligence will play an important role in the freshness monitoring sensors. Users can form a network of real-time monitoring and data analytics systems to easily monitor the status of their food products via mobile devices or smart homes. Secondly, future developments in sensor technology will focus on multifunctional integration, with the ability to detect multiple indicators of spoilage (e.g., temperature, humidity, volatile compounds) simultaneously. This will make sensors more efficient and accurate in complex food monitoring environments. Finally, with the development of flexible printing and nanotechnology, the manufacturing cost of sensors will be further reduced to support large-scale commercialization and popularization. Low-cost, environmentally friendly, and renewable sensor materials will become an important direction for future research, further promoting the popularization and protection of food safety.

However, several challenges must be addressed to realize these prospects. First, enhancing the stability and reliability of sensors, particularly for long-term use in complex environments, is crucial. Sensors need to maintain consistent performance over extended periods without being affected by environmental factors. Second, standardizing sensor data is essential for ensuring that readings are comparable and reliable across different systems and applications. This standardization will facilitate broader adoption and integration into existing food safety protocols. Lastly, achieving cost control and mass production capabilities is necessary for the industrialization of sensor technology. Making these sensors affordable and scalable will be key to their widespread adoption in the food industry.

## 6. Conclusions

In this review, we outline the major advancements in sensor technologies for assessing the freshness of aquatic products. Over recent years, the most widely utilized sensor devices for this purpose have been artificial senses and biosensors, including electronic noses and tongues. However, issues such as limited sensitivity and sensor aging remain significant challenges for artificial senses. Moreover, their stability and robustness can be adversely affected in complex environments. With the development of flexible materials and fabrication techniques, flexible sensors have emerged as an innovative solution for assessing the freshness of aquatic products, owing to their superior mechanical properties and wearable features. Consequently, these technologies are flourishing and demonstrating considerable potential and benefits. In summary, with the ongoing advancements in instrumentation and computer science, the application of modern advanced sensor techniques for aquatic products in cold chain logistics is expected to expand significantly in the future. 

## Figures and Tables

**Table 1:** Comparison of various pattern recognition methods for aquatic products freshness assessment using E-nose detection.

Method	Mechanism	Accuracy	Sensitivity	Advantages	Disadvantages	Reference
Principal Component Analysis (PCA)	Statistical technique for dimensionality reduction and data pattern recognition	91%	90.9%	Simple data processing, good correlation	Sensitive to noise, requires large sample data	[67]
Convolutional Neural Network (CNN)	Deep learning model for hierarchical pattern recognition	92.31%	91.5%	Highly nonlinear modeling, high prediction accuracy	Computationally complex, long training time	[69]
Laplacian Eigenmap (LE)	Nonlinear dimensionality reduction method using graph-based techniques	85%	84.1%	Can handle nonlinear data	High algorithmic complexity	[70]
Ensemble-based Support Vector Machine (SVM)	Combines multiple SVM classifiers for robust decision-making	84.1%	72.7%	Effective for high-dimensional data	Sensitive to parameter selection, computationally expensive	[72]
Ultra-Fast Gas Chromatography (UFGC)	Separates and analyzes volatile compounds using high-speed gas chromatography	89%	88%	Fast speed, high resolution	Expensive equipment, complex operation	[71]

**Table 2:** Comparison of various advance sensor technologies.

Advanced Sensor Technology	Advantages	Disadvantages	Application
Electronic Nose	Short response time, fast monitoring, simple pre-processing, wide evaluation range, good repeatability	Susceptible to environmental interference, high equipment cost	Rapid detection of odor in food products
Electronic Tongue	Responds to some low concentrations of substances generated in the early stages	Susceptible to environmental interference, high equipment cost	Rapid detection of taste in food
Biosensors	High sensitivity and specificity	Poor stability, not suitable for precise biochemical analysis	Sensitive biochemical analysis in controlled conditions
Flexible Sensors	Flexibility, multi-functional integration, suitable for real-time and continuous monitoring	Currently facing challenges in stability and long-term use in complex environments	Real-time freshness detection and continuous monitoring in aquatic products

## References

[1] Ren F. (2022). The Development Model of Japanese Cold Chain Logistics: The Example of Seafood Transportation. Asian Bus. Res..

[2] Liu K., Gao W., Cong C. (2023). Oyster cold chain logistics monitoring system based on RFID and cloud platforms. SPIE.

[3] Zhou H. (2022). Application of RFID Information Technology in Fresh Food Cold Chain Logistics Management. J. Phys. Conf. Ser..

[4] Ye B., Chen J., Fu L., Wang Y. (2022). Application of nondestructive evaluation (NDE) technologies throughout cold chain logistics of seafood: Classification, innovations and research trends. LWT.

[5] Huang W.-Y., Xia J., Wang Y., Jin X., Zhu H., Zhang X. (2024). Flexible Multimode Sensors Based on Hierarchical Microstructures Enable Non-Destructive Grading of Fruits in Cold Chain Logistics. Mater. Today Sustain..

[6] Zhou L., Li Q.-S., Li F., Jin C. (2022). Research on Green Technology Path of Cold-Chain Distribution of Fresh Products Based on Sustainable Development Goals. Sustainability.

[7] Guney S., Atasoy A. Fish Freshness Assessment by Using Electronic Nose. Proceedings of the 2013 36th International Conference on Telecommunications and Signal Processing (TSP).

[8] Apetrei I., Rodriguez-Mendez M.L., Apetrei C., de Saja J.A. (2013). Fish Freshness Monitoring Using an E-Tongue Based on Polypyrrole Modified Screen-Printed Electrodes. IEEE Sens. J..

[9] Han F., Huang X., Teye E., Gu F., Gu H. (2014). Nondestructive detection of fish freshness during its preservation by combining electronic nose and electronic tongue techniques in conjunction with chemometric analysis. Anal. Methods.

[10] Wijaya D., Syarwan N.F., Nugraha M.A., Ananda D., Fahrudin T., Handayani R. (2023). Seafood Quality Detection Using Electronic Nose and Machine Learning Algorithms With Hyperparameter Optimization. IEEE Access.

[11] Munekata P.E., Finardi S., de Souza C.K., Meinert C., Pateiro M., Hoffmann T., Domínguez R., Bertoli S., Kumar M., Lorenzo J. (2023). Applications of Electronic Nose, Electronic Eye and Electronic Tongue in Quality, Safety and Shelf Life of Meat and Meat Products: A Review. Sensors.

[12] Hou Y., Genua M., Batista D.T., Calemczuk R., Buhot A., Fornarelli P., Koubachi J., Bonnaffé D., Saesen E., Laguri C. (2012). Continuous evolution profiles for electronic-tongue-based analysis. Angew. Chem. Int. Ed..

[13] Wang P., Zhuang L., Zhen Q., Gao K., Gao Z.B. (2021). Research Progress of Bioinspired Smell and Taste Sensors. J. Appl. Chem..

[14] Gui X., Li H., Ma R., Tian L.-Y., Hou F., Li H., Fan X.-H., Wang Y., Yao J., Shi J. (2023). Authenticity and species identification of Fritillariae cirrhosae: A data fusion method combining electronic nose, electronic tongue, electronic eye and near infrared spectroscopy. Front. Chem..

[15] Li H., Wang P., Lin Z.-Z., Wang Y.-L., Gui X., Fan X.-H., Dong F.-Y., Zhang P.-P., Li X., Liu R. (2024). Identification of Bletilla striata and related decoction pieces: A data fusion method combining electronic nose, electronic tongue, electronic eye, and high-performance liquid chromatography data. Front. Chem..

[16] Fu B., Fang C., Li Z., Zeng Z., He Y., Chen S., Yang H. (2024). The Effect of Heat Stress on Sensory Properties of Fresh Oysters: A Comprehensive Study Using E-Nose, E-Tongue, Sensory Evaluation, HS–SPME–GC–MS, LC–MS, and Transcriptomics. Foods.

[17] Ventura-Aguilar R., Lucas-Bautista J.A., Árevalo-Galarza M.L., Bosquez-Molina E. (2024). Volatile Organic Compounds as a Diagnostic Tool for Detecting Microbial Contamination in Fresh Agricultural Products: Mechanism of Action and Analytical Techniques. Processes.

[18] Chaudhary D., Upadhyay J.B., Koshta V. (2015). Application of biosensors in dairy-food industry. J. Food Qual..

[19] Wu C., Sun J., Chen M., Ge Y., Ma J., Hu Y., Pang J., Yan Z. (2019). Effect of oxidized chitin nanocrystals and curcumin into chitosan films for seafood freshness monitoring. Food Hydrocoll..

[20] Zhao C., Shen J., Xu S., Wei J., Liu H., Xie S., Pan Y., Zhao Y., Zhu Y. (2022). Ultra-efficient trimethylamine gas sensor based on Au nanoparticles sensitized WO3 nanosheets for rapid assessment of seafood freshness. Food Chem..

[21] Schöning M., Poghossian A. (2022). Recent advances in biologically sensitive field-effect transistors (BioFETs). Anal. Chem..

[22] Chen X. (2022). Research progress in several types of fish freshness rapid detection methods. Food Sci. Technol..

[23] Liu Y., Wang C., Yuan X., Xiong T. Research on the Safety of Sea Crab Based on Machine Olfactory. Proceedings of the 2020 13th International Symposium on Computational Intelligence and Design (ISCID).

[24] Zhu C., Zhou T., Xia H., Zhang T. (2023). Flexible Room-Temperature Ammonia Gas Sensors Based on PANI-MWCNTs/PDMS Film for Breathing Analysis and Food Safety. Nanomaterials.

[25] Yang Y., Zheng X., Liu J., Ye C., Wan B. (2023). Semiconductor-Type Triethylamine Sensor for Food Detection Based on WO_3_ Nanomaterials. J. Electrochem. Soc..

[26] Tseng S.-Y., Li S.-Y., Yi S.-Y., Sun A.Y., Gao D.-Y., Wan D. (2017). Food Quality Monitor: Paper-Based Plasmonic Sensors Prepared Through Reversal Nanoimprinting for Rapid Detection of Biogenic Amine Odorants. ACS Appl. Mater. Interfaces.

[27] Shen J., Xu S., Zhao C., Qiao X., Liu H., Zhao Y., Wei J., Zhu Y. (2021). Bimetallic Au@Pt Nanocrystal Sensitization Mesoporous α-Fe_2_O_3_; Hollow Nanocubes for Highly Sensitive and Rapid Detection of Fish Freshness at Low Temperature. ACS Appl. Mater. Interfaces.

[28] Jia R., Tian W., Bai H., Zhang J., Wang S., Zhang J. (2019). Amine-responsive cellulose-based ratiometric fluorescent materials for real-time and visual detection of shrimp and crab freshness. Nat. Commun..

[29] Han K., Jiang B., Tong Y., Zhang W., Zou X., Shi J., Su X. (2023). Flexible-fabricated sensor module with programmable magnetic actuators coupled to L-cysteine functionalized Ag@Fe_3_O_4_ complexes for Cu^2+^ detection in fish tissues. Biomed. Microdevices.

[30] Andre R.S., Schneider R., DeLima G.R., Fugikawa-Santos L., Corrêa D. (2024). Wireless Sensor for Meat Freshness Assessment Based on Radio Frequency Communication. ACS Sens..

[31] Zhang Z., Sun Y., Sang S., Jia L., Ou C. (2022). Emerging Approach for Fish Freshness Evaluation: Principle, Application and Challenges. Foods.

[32] Zou T., Liu Z. (2024). Recent advances in preservation and freshness monitoring methods for fish: A review on the quality and structural changes of fish after slaughter. Adv. Fish Technol..

[33] Botta J.R. (1995). Evaluation of Seafood Freshness Quality.

[34] Cheng J.-H., Sun D.-W., Zeng X., Liu D. (2015). Recent Advances in Methods and Techniques for Freshness Quality Determination and Evaluation of Fish and Fish Fillets: A Review. Crit. Rev. Food Sci. Nutr..

[35] El-Shamery GE M. (2023). Studies on Microbial, Physical and Chemical Quality of Fresh Yemeni Rabbit Meats During Storage in Taiz City. Taiz Univ. J. Nat. Appl. Sci..

[36] Cheng J.-H., Sun D.-W., Pu H., Zhu Z. (2015). Development of hyperspectral imaging coupled with chemometric analysis to monitor K value for evaluation of chemical spoilage in fish fillets. Food Chem..

[37] Tang Q., Zou Z., Huang Y., Liang S., Li H., Xu L. (2023). Novel ammonia-responsive carboxymethyl cellulose/Co-MOF multifunctional films for real-time visual monitoring of seafood freshness. Int. J. Biol. Macromol..

[38] Kannan S.K., Ambrose B., Sudalaimani S., Pandiaraj M., Giribabu K., Kathiresan M. (2020). A review on chemical and electrochemical methodologies for the sensing of biogenic amines. Anal. Methods.

[39] Dehaut A., Duthen S., Grard T., Krzewinski F., N’Guessan A., Brisabois A., Duflos G. (2016). Development of an SPME-GC-MS method for the specific quantification of dimethylamine and trimethylamine: Use of a new ratio for the freshness monitoring of cod fillets. J. Sci. Food Agric..

[40] Pacquit A., Lau K., Diamond D. (2004). Development of a colorimetric sensor for monitoring of fish spoilage amines in packaging headspace. IEEE Sens..

[41] Zhou C., Sun D.-W., Ma J., Qin A., Tang B., Lin X.-R., Cao S.-L. (2024). Assembly-Induced Emission of Copper Nanoclusters: Revealing the Sensing Mechanism for Detection of Volatile Basic Nitrogen in Seafood Freshness On-Site Monitoring. ACS Appl. Mater. Interfaces.

[42] Hassoun A., Karoui R. (2015). Quality evaluation of fish and other seafood by traditional and nondestructive instrumental methods: Advantages and limitations. Crit. Rev. Food Sci. Nutr.

[43] Horsfall Jnr M., Gentleman P.C., Adowei P., Dikio E.D. (2023). Evaluation of total volatile bases and trimethylamine in hake (*Merluccius capensis*) fish preserved at low temperature in Vanderbijlpark, South Africa. World J. Adv. Res. Rev..

[44] Taliadourou D., Papadopoulos V.D., Domvridou E., Savvaidis I., Kontominas M. (2003). Microbiological, chemical and sensory changes of whole and filleted Mediterranean aquacultured sea bass (*Dicentrarchus labrax*) stored in ice. J. Sci. Food Agric..

[45] Franceschelli L., Berardinelli A., Dabbou S., Ragni L., Tartagni M. (2021). Sensing Technology for Fish Freshness and Safety: A Review. Sensors.

[46] Özkaya P., Dağbağlı S. (2021). Usage of Natural Colour Indicators in Packaging Materials for Monitorization of Meat Freshness. Turk. J. Agric.—Food Sci. Technol..

[47] Athauda T., Karmakar N.C. (2019). Review of RFID-based sensing in monitoring physical stimuli in smart packaging for food-freshness applications. Wirel. Power Transf..

[48] Ma P., Xu W., Teng Z., Luo Y., Gong C., Wang Q. (2022). An Integrated Food Freshness Sensor Array System Augmented by a Metal-Organic Framework Mixed-Matrix Membrane and Deep Learning. ACS Sens..

[49] Dong Y., Sun W., Li W., Li L., Xia F., Wu L., Jin Y., Saldaña M.A., Sun W. (2023). Poly-L-lactic acid/lead(II) acetate basic colour indicator membrane for visual monitoring in shrimp freshness. Packag. Technol. Sci..

[50] Wang F., Qiu L., Tian Y. (2021). Super Anti-Wetting Colorimetric Starch-Based Film Modified with Poly(dimethylsiloxane) and Micro-/Nano-Starch for Aquatic-Product Freshness Monitoring. Biomacromolecules.

[51] Luo X., Zaitoon A., Lim L. (2022). A review on colorimetric indicators for monitoring product freshness in intelligent food packaging: Indicator dyes, preparation methods, and applications. Crit. Rev. Food Sci. Nutr..

[52] Chen M.-M., Li B.-H., Wu Y., He Z., Xiong X.-B., Han W.-D., Liu B., Yang S. (2023). Intelligent biogenic pH-sensitive and amine-responsive color-changing label for real-time monitoring of shrimp freshness. J. Sci. Food Agric..

[53] Devarayan K., Palanisamy Y., Mohan G., Theivasigamani A., Kandasamy S., Sekar V., John E.U.S., Sukumaran M., Marimuthu R., Anjappan H. (2024). Non-invasive measurement of spoilage of packed fish using halochromic sensor. Pigment Resin Technol..

[54] Ghozzi K., Nakbi A., Challouf R., Dhiab R. (2023). A review on microbial contamination cases in Tunisian coastal marine areas. Water Sci. Technol..

[55] Moosavi-Nasab M., Khoshnoudi-Nia S. (2021). Combining Knowledge- and Data-driven Fuzzy Approach to Evaluate Shelf-life of Various Seafood Products. Food Qual. Saf..

[56] Amelin V.G., Shogah Z.A., Bolshakov D., Tretyakov A.V., Nesterenko I.S., Kish L. (2023). Determination of seafood spoilage by digital colorimetry of indicator test systems. J. Anal. Chem..

[57] Wu D., Zhang M., Chen H., Bhandari B. (2020). Freshness monitoring technology of fish products in intelligent packaging. Crit. Rev. Food Sci. Nutr..

[58] He Y., Yuan Y., Gao Y., Chen M., Li Y., Zou Y., Liao L., Li X., Wang Z., Li J. (2024). Enhancement of Colorimetric pH-Sensitive Film Incorporating Amomum tsao-ko Essential Oil as Antibacterial for Mantis Shrimp Spoilage Tracking and Fresh-Keeping. Foods.

[59] Anisimov D.S., Abramov A.A., Gaidarzhi V.P., Kaplun D.S., Agina E.V., Ponomarenko S.A. (2023). Food Freshness Measurements and Product Distinguishing by a Portable Electronic Nose Based on Organic Field-Effect Transistors. ACS Omega.

[60] Meléndez F., Arroyo P., Gómez-Suárez J., Palomeque-Mangut S., Suárez J., Lozano J. (2022). Portable Electronic Nose Based on Digital and Analog Chemical Sensors for 2,4,6-Trichloroanisole Discrimination. Sensors.

[61] Radi R., Wahyudi E., Adhityamurti M.D., Putro JP L.Y., Barokah B., Rohmah D.N. (2021). Freshness assessment of tilapia fish in traditional market based on an electronic nose. Bull. Electr. Eng. Inform..

[62] Sánchez R., Alejo M., Escribano P., Arroyo P., Meléndez F., Lozano J. (2024). Classification of Fish Freshness and Prediction of Mesophilic Aerobic Microbial Count with an Electronic Nose. IEEE Sens. J..

[63] Grassi S., Benedetti S., Opizzio M., Nardo E.D., Buratti S. (2019). Meat and Fish Freshness Assessment by a Portable and Simplified Electronic Nose System (Mastersense). Sensors.

[64] Wang M., Gao F., Wu Q., Zhang J., Xue Y., Wan H., Wang P. (2018). Real-time assessment of food freshness in refrigerators based on a miniaturized electronic nose. Anal. Methods.

[65] Alloyarova Y. (2023). Application of the “electronic nose” for evaluating volatile compounds of semi-finished small fish. J. Food Process. Preserv..

[66] Madhubhashini M.N., Liyanage C.P., Alahakoon A., Liyanage R. (2024). Development of a comprehensive classification model for determining the storage day of frigate tuna (*Auxis thazard*) for freshness evaluation using a portable electronic nose. Int. J. Food Sci. Technol..

[67] Gholamhosseini H., Luo D., Liu H., Xu G. (2007). Intelligent Processing of E-nose Information for Fish Freshness Assessment. IEEE Sens. J..

[68] Tian X.-Y., Cai Q., Zhang Y.-M. (2011). Rapid Classification of Hairtail Fish and Pork Freshness Using an Electronic Nose Based on the PCA Method. Sensors.

[69] Sun Y., Zhang X. (2022). Tilapia freshness prediction utilizing gas sensor array system combined with convolutional neural network pattern recognition model. Int. J. Food Sci. Technol..

[70] Zhu P., Chen C.-S., Xu B., Lu M. (2016). Research on Freshness Detection for Chinese Mitten Crab Based on Machine Olfaction. Lect. Notes Comput. Sci..

[71] Weng X., Luan X., Kong C., Chang Z., Li Y., Zhang S., Al-Majeed S., Xiao Y. (2020). A Comprehensive Method for Assessing Meat Freshness Using Fusing Electronic Nose, Computer Vision, and Artificial Tactile Technologies. J. Sens..

[72] Mohareb F., Papadopoulou O.S., Panagou E.Z., Nychas G.E., Bessant C. (2016). Ensemble-based support vector machine classifiers as an efficient tool for quality assessment of beef fillets from electronic nose data. Anal. Methods.

[73] Burgués J., Esclapez M., Doñate S., Marco S. (2021). RHINOS: A lightweight portable electronic nose for real-time odor quantification in wastewater treatment plants. iScience.

[74] Wei X., Zhang M., Chen K., Huang M., Mujumdar A., Yang C. (2024). Intelligent detection and control of quality deterioration of fresh aquatic products in the supply chain: A review. Comput. Electron. Agric..

[75] Huang Y., Ren X., Wang Y., Sun D., Xu L., Wu F. Electronic Nose System implemented on ZYNQ Platform for Fruits Freshness Classification. Proceedings of the 2023 IEEE 6th Information Technology, Networking, Electronic and Automation Control Conference, ITNEC.

[76] Chen J., Gu J., Zhang R., Mao Y., Tian S. (2019). Freshness Evaluation of Three Kinds of Meats Based on the Electronic Nose. Sensors.

[77] Xiong Y., Li Y., Wang C., Shi H., Wang S., Yong C., Gong Y., Zhang W., Zou X. (2023). Non-Destructive Detection of Chicken Freshness Based on Electronic Nose Technology and Transfer Learning. Agriculture.

[78] Feng H., Zhang M., Liu P., Liu Y., Zhang X. (2020). Evaluation of IoT-Enabled Monitoring and Electronic Nose Spoilage Detection for Salmon Freshness During Cold Storage. Foods.

[79] Kaya Z., Koca I. (2020). Electronic Tongue Applications in Food Engineering. Turk. J. Agric.—Food Sci. Technol..

[80] Cho S., Moazzem M. (2022). Recent Applications of Potentiometric Electronic Tongue and Electronic Nose in Sensory Evaluation. Korean J. Food Sci. Anim. Resour..

[81] Li Y., Lei J., Liang D. (2021). Identification of Fake Green Tea by Sensory Assessment and Electronic Tongue. Food Sci. Technol. Res..

[82] Piccinin A.C.V., Coatrini-Soares A., Franco G.T., Bondancia T.J., Coatrini-Soares J., Oliveira O.N., Mattoso L.H. (2024). Electronic tongue made of gelatin self-supporting films on printed electrodes to detect lactose. Front. Sens..

[83] Duan X., Li Z., Wang L., Lin H., Wang K. (2022). Engineered nanomaterials-based sensing systems for assessing the freshness of meat and aquatic products: A state-of-the-art review. Compr. Rev. Food Sci. Food Saf..

[84] Nowshad F., Khan M. (2021). Electronic Tongue for Food Safety and Quality Assessment. Nondestructive Testing of Food Quality.

[85] Waimin J., Gopalakrishnan S., Heredia-Rivera U., Kerr N.A., Nejati S., Gallina N.L., Bhunia A., Rahimi R. (2022). Low-Cost Nonreversible Electronic-Free Wireless pH Sensor for Spoilage Detection in Packaged Meat Products. ACS Appl. Mater. Interfaces.

[86] Cong H., Ding H., Wang G., Wang X., Chen L. (2023). Smartphone-assisted multicolor hypoxanthine sensing for on-site freshness assessment of aquatic products. Sens. Actuators B Chem..

[87] Nam S., Lee J., Kim E.-J., Koo J., Shin Y., Hwang T. (2023). Electronic tongue for the simple and rapid determination of taste and odor compounds in water. Chemosphere.

[88] Tomita S. (2022). Chemical Tongues: Biomimetic Recognition Using Arrays of Synthetic Polymers. Polym. J..

[89] Cetó X., del Valle M. (2022). Electronic tongue applications for wastewater and soil analysis. iScience.

[90] Chang C.-C., Saad B., Surif M., Ahmad M.N., Shakaff A.Y.M. (2008). Disposable E-Tongue for the Assessment of Water Quality in Fish Tanks. Sensors.

[91] Podrażka M., Baczynska E., Kundys M., Jeleń P.S., Witkowska Nery E. (2018). Electronic Tongue—A Tool for All Tastes?. Biosensors.

[92] Ghrissi H., Veloso A.C., Marx I.M.G., Dias T., Peres A.M. (2021). A Potentiometric Electronic Tongue as a Discrimination Tool of Water-Food Indicator/Contamination Bacteria. Chemosensors.

[93] Thakur D., Pandey C.M., Kumar D. (2022). Highly Sensitive Enzymatic Biosensor Based on Polyaniline-Wrapped Titanium Dioxide Nanohybrid for Fish Freshness Detection. Appl. Biochem. Biotechnol..

[94] Xu X., Wu X., Zhuang S., Zhang Y., Ding Y., Zhou X. (2022). Colorimetric Biosensor Based on Magnetic Enzyme and Gold Nanorods for Visual Detection of Fish Freshness. Biosensors.

[95] Li J., Zhang N., Yang X., Yang X., Wang Z., Liu H.-M. (2022). RhB@MOF-5 Composite Film as a Fluorescence Sensor for Detection of Chilled Pork Freshness. Biosensors.

[96] Abbasi-Moayed S., Orouji A., Hormozi-Nezhad M. (2023). Multiplex Detection of Biogenic Amines for Meat Freshness Monitoring Using Nanoplasmonic Colorimetric Sensor Array. Biosensors.

[97] Sriramulu G., Verma R., Singh K.R.B., Singh P., Chakra C., Mallick S., Singh R.P., Sadhana K., Singh J. (2024). Self-assembled Copper Oxide Nanoflakes for Highly Sensitive Electrochemical Xanthine Detection in Fish-Freshness Biosensors. J. Mol. Struct..

[98] Karakuş S., Baytemir G., Taşaltın N. (2022). Digital Colorimetric and Non-Enzymatic Biosensor with Nanoarchitectonics of Lepidium Meyenii-Silver Nanoparticles and Cotton Fabric: Real-Time Monitoring of Milk Freshness. Appl. Phys. A.

[99] Su X., Sutarlie L., Loh X. (2020). Sensors, Biosensors, and Analytical Technologies for Aquaculture Water Quality. Biosensors.

[100] Forinová M., Seidlová A., Pilipenco A., Lynn N.S., Obořilová R., Farka Z., Skládal P., Saláková A., Spasovová M., Houska M. (2023). A Comparative Assessment of a Piezoelectric Biosensor Based on a New Antifouling Nanolayer and Cultivation Methods: Enhancing *S. aureus* Detection in Fresh Dairy Products. Curr. Res. Biotechnol..

[101] Wang Z., Ma B., Shen C., Lai O., Tan C., Cheong L. (2019). Electrochemical Biosensing of Chilled Seafood Freshness by Xanthine Oxidase Immobilized on Copper-Based Metal–Organic Framework Nanofiber Film. J. Food Sci..

[102] Liu B., Gurr P., Qiao G. (2020). An irreversible spoilage sensor for protein-based food. ACS Sens..

[103] Luo X., Lim L. (2020). An Inkjet-Printed Sulfonephthalein Dye Indicator Array for Volatile Amine Detection. J. Food Sci..

[104] Milintha Mary T.P., Kumaravel B., Nagamaniammai G., Karishma S., Essa M.M., Qoronfleh M.W., Chacko L. (2023). Biosensors as Freshness Indicator for Packed Animal and Marine Products: A Review. Int. Food Res. J..

[105] González-Martín A., Lewis B., Raducanu M., Kim J. (2010). An Array-Based Sensor for Seafood Freshness Assessment. Bull. Korean Chem. Soc..

[106] Majer-Baranyi K., Székács A., Adányi N. (2023). Application of Electrochemical Biosensors for Determination of Food Spoilage. Biosensors.

[107] Ma Q., Lu X., Wang W., Hubbe M., Liu Y., Mu J., Wang J., Sun J., Rojas O. (2021). Recent Developments in Colorimetric and Optical Indicators Stimulated by Volatile Base Nitrogen to Monitor Seafood Freshness. Food Packag. Shelf Life.

[108] Sheng W., Sun C., Fang G.-z., Wu X., Hu G., Zhang Y., Wang S. (2016). Development of an Enzyme-Linked Immunosorbent Assay for the Detection of Tyramine as an Index of Freshness in Meat and Seafood. J. Agric. Food Chem..

[109] Chen J., Zhang J., Liu C., Sun Y., Han X., Sun X., Pei X., Huang F., Li X., Chen A. (2024). Development of Halochromic Labels Based on Binary Systems of Cationic Guar Gum and κ-Carrageenan Loaded with Alizarin Red S for Monitoring Milk and Seafood Freshness. Food Hydrocoll..

[110] Mary S.K., Koshy R.R., Daniel J., Koshy J., Pothen L., Thomas S. (2020). Development of Starch Based Intelligent Films by Incorporating Anthocyanins of Butterfly Pea Flower and TiO2 and Their Applicability as Freshness Sensors for Prawns During Storage. RSC Adv..

[111] Endo H., Wu H. (2019). Biosensors for the Assessment of Fish Health: A Review. Fish. Sci..

[112] Chouler J., Monti M.D., Morgan W.J., Cameron P.J., Di Lorenzo M. (2019). A Photosynthetic Toxicity Biosensor for Water. Electrochim. Acta.

[113] Xiong X., Tan Y., Mubango E., Shi C., Regenstein J.M., Yang Q., Hong H., Luo Y. (2022). Rapid Freshness and Survival Monitoring Biosensors of Fish: Progress, Challenge, and Future Perspective. Trends Food Sci. Technol..

[114] Kolarevic J., Calduch-Giner J., Espmark Å.M., Evensen T., Sosa J., Pérez-Sánchez J. (2021). A Novel Miniaturized Biosensor for Monitoring Atlantic Salmon Swimming Activity and Respiratory Frequency. Animals.

[115] Huang W., Xia J., Wang X., Zhao Q., Zhang M., Zhang X. (2023). Improvement of Non-Destructive Detection of Lamb Freshness Based on Dual-Parameter Flexible Temperature-Impedance Sensor. Food Control.

[116] Li X., Jin L., Ni A., Zhang L., He L., Gao H., Lin P., Zhang K., Chu X., Wang S. (2022). Tough and Antifreezing MXene@Au Hydrogel for Low-Temperature Trimethylamine Gas Sensing. ACS Appl. Mater. Interfaces.

[117] Quan W., Shi J., Luo H., Fan C., Lv W., Chen X., Zeng M., Yang J., Hu N., Su Y. (2023). Fully Flexible MXene-Based Gas Sensor on Paper for Highly Sensitive Room-Temperature Nitrogen Dioxide Detection. ACS Sens..

[118] Bai R., Gao Y., Wu R., Ju K., Tan J., Xuan F. (2021). Laser Direct Writing of Flexible Sensor Arrays Based on Carbonized Carboxymethylcellulose and Its Composites for Simultaneous Mechanical and Thermal Stimuli Detection. ACS Appl. Mater. Interfaces.

[119] Huang Y., Jiao W., Chu Z., Nie X., Wang R., He X. (2020). SnS2 Quantum Dots Based Optoelectronic Flexible Sensor for Ultrasensitive Detection of NO2 Down to One-ppb. ACS Appl. Mater. Interfaces.

[120] Hu X., Zhang X., Li Y., Shi J., Huang X., Li Z., Zhang J., Li W., Xu Y., Zou X. (2022). Easy-to-Use Visual Sensing System for Milk Freshness, Sensitized with Acidity-Responsive N-Doped Carbon Quantum Dots. Foods.

[121] Mahajan P., Luca A., Edelenbos M. (2016). Development of a Small and Flexible Sensor-Based Respirometer for Real-Time Determination of Respiration Rate, Respiratory Quotient and Low O2 Limit of Fresh Produce. Comput. Electron. Agric..

[122] Yılmazoğlu E. (2024). Digital Image Colorimetric Detection of H2O2 Utilizing PEG/Ag/AgO Nanoparticles Derived from Tangerine Leaf Extract. J. Turk. Chem. Soc. Sect. A Chem..

[123] Mestry S., Satalkar V., Mhaske S.T. (2023). Development of Imine-Azo-Dyes Derived from Vanillin and Salicylaldehyde for pH-Sensing in Smart Packaging. Pigment Resin Technol..

[124] Dudnyk I., Janeček E.-R., Vaucher-Joset J., Stellacci F. (2018). Edible Sensors for Meat and Seafood Freshness. Sens. Actuators B Chem..

[125] Wu H., Liu H., Xing D., Li F., Chen Q., Jiang W. (2023). Design of Seafood Freshness Detector Based on Trimethylamine Gas Measurement. J. Phys. Conf. Ser..

[126] Konoplev G., Sünter A., Kuznetsov A., Frorip A., Korsakov V., Stepanova O., Lyalin D., Stepanova O.V. (2023). Assessment of the Freshness of Fish and Poultry Meat by Fast Protein and Metabolite Liquid Chromatography Using a New Optical Sensor. Proc. Int. Electron. Conf. Biosens..

[127] Li S., Chen S., Zhuo B., Li Q., Liu W., Guo X. (2017). Flexible Ammonia Sensor Based on PEDOT Silver Nanowire Composite Film for Meat Freshness Monitoring. IEEE Electron Device Lett..

[128] Zhang D., Zhou D., Mi H., Wang Z., Zhang P., Xi G. (2024). Highly Sensitive Trimethylamine QCM Sensor Based on Porous Functionalized Tungsten Disulfide/Polyacrylic Acid Composite for Seafood Freshness Detection. Sens. Actuators B Chem..

[129] Zeng Q., Wang Y., Javeed A., Chen F., Li J., Guan Y., Chen B., Han B. (2024). Preparation and Properties of Polyvinyl Alcohol/Chitosan-Based Hydrogel with Dual pH/NH3 Sensor for Naked-Eye Monitoring of Seafood Freshness. Int. J. Biol. Macromol..

[130] Choi J., Visagie I., Chen Y., Abbel R., Parker K. (2023). NFC-Enabled Dual-Channel Flexible Printed Sensor Tag. Sensors.

[131] Kim J., Yoo H., Pham Ba V.A., Shin N., Hong S. (2018). Dye-Functionalized Sol-Gel Matrix on Carbon Nanotubes for Refreshable and Flexible Gas Sensors. Sci. Rep..

[132] Mu B., Dong Y., Qian J., Wang M., Yang Y., Nikitina M.A., Zhang L., Xiao X. (2022). Hydrogel Coating Flexible pH Sensor System for Fish Spoilage Monitoring. Mater. Today Chem..

[133] Gopalakrishnan S., Nejati S., Sedaghat S., Gupta K., Mishra R., Rahimi R. (2023). Electronic-Free Low-Cost Wireless Sensor Tag for Monitoring Fish Freshness. Sens. Actuators B Chem..

[134] Feng H., Fu Y., Huang S., Glamuzina B., Zhang X. (2023). Novel Flexible Sensing Technology for Nondestructive Detection on Live Fish Health/Quality During Waterless and Low-Temperature Transportation. Biosens. Bioelectron..

[135] Damdam A., Ozay L., Ozcan C., Alzahrani A., Helabi R., Salama K. (2023). IoT-Enabled Electronic Nose System for Beef Quality Monitoring and Spoilage Detection. Foods.

[136] Felicia W.X.L., Rovina K., Nur’ Aqilah N.M., Vonnie J., Yin K.W., Huda N. (2023). Assessing Meat Freshness via Nanotechnology Biosensors: Is the World Prepared for Lightning-Fast Pace Methods?. Biosensors.

[137] Vu C., Lin Y., Haenen S.R.R., Marschall J., Hummel A., Wouters S.F.A., Raats J., de Jong A.D., Yan J., Prins M. (2023). Real-Time Immunosensor for Small-Molecule Monitoring in Industrial Food Processes. Anal. Chem..

[138] Bao F., Liang Z., Deng J., Lin Q., Li W., Peng Q., Fang Y. (2022). Toward Intelligent Food Packaging of Biosensor and Film Substrate for Monitoring Foodborne Microorganisms: A Review of Recent Advancements. Crit. Rev. Food Sci. Nutr..

[139] Gu R., Duan Y., Li Y., Luo Z. (2023). Fiber-Optic-Based Biosensor as an Innovative Technology for Point-of-Care Testing Detection of Foodborne Pathogenic Bacteria To Defend Food and Agricultural Product Safety. J. Agric. Food Chem..

[140] Li S., Tang W., Chen S., Si Y., Liu R., Guo X. (2023). Flexible Organic Polymer Gas Sensor and System Integration for Smart Packaging. Adv. Sens. Res..

[141] Xia J., Huang W.-Y., Majer-Baranyi K., Zhang M., Zhang X. (2023). Conformal Temperature/Impedance Sensing Patch Based on Graphene Materials for Nondestructive Detection of Fish Freshness. ACS Appl. Mater. Interfaces.

